# ω9 Monounsaturated and Saturated Colostrum Fatty Acids May Benefit Newborns in General and Subtle Hypothyroid Stages

**DOI:** 10.3390/nu17122017

**Published:** 2025-06-17

**Authors:** Meric A. Altinoz, Muhittin A. Serdar, Selim M. Altinoz, Mustafa Eroglu, Murat Muhcu, Pinar Kumru, Aysel Ozpinar

**Affiliations:** 1 Department of Medical Biochemistry, Acibadem University, 34638 Istanbul, Turkey; adilmeric.altinoz@saglik.gov.tr (M.A.A.); muhittin.serdar@acibadem.edu.tr (M.A.S.); 2 Bachelor Student of Statistics, Yildiz Technical University, 34220 Istanbul, Turkey; mustafa.altinoz@std.yildiz.edu.tr; 3 Istanbul Zeynep Kamil Kadın ve Çocuk Hastalıkları Sağlık Uygulama ve Araştırma Merkezi, 34638 Istanbul, Turkey; mustafa.eroglu@sbu.edu.tr (M.E.); pinar.kumru@sbu.edu.tr (P.K.); 4 Istanbul Ümraniye Sağlık Uygulama ve Araştırma Merkezi, 34638 Istanbul, Turkey; murat.muhcu@sbu.edu.tr

**Keywords:** colostrum, ω9 fatty acids, erucic acid, thyroid hormones

## Abstract

**Objectives:** This study analyzed correlations of colostrum fatty acids (FAs), newborns’ and mothers’ thyroid hormones (THs), and birth weight, all crucially important in neonatal health. **Methods:** LC-MS/MS was used to measure 22 FAs in the colostrum of 78 healthy mothers who delivered term babies. FT3, FT4, and TSH levels were determined in the mothers’ serum, and newborns’ TSH was measured in heel-pricked specimens. Correlations were defined in the whole cohort and the subsets, which were separated according to ranges of birth weight, thyroid hormones, and mothers’ body mass index. Phyton Software was used for statistics. **Results:** The colostrum’s total FA content was highly variable and correlated positively with the percentage values of arachidic, gondoic, and nervonic acids. Five FAs all positively correlated with birth weight for the entire cohort—including ω9 gondoic, erucic, and nervonic acids as well as saturated behenic and lignoceric acids—all produced with the same elongases. These correlations were relevant to gondoic, nervonic, behenic, and lignoceric acids when mothers with low FT4 levels were evaluated separately and to erucic acid in the subset comprising mothers with high TSH values. **Conclusions:** The priming of breast epithelia to adjust the colostrum quality starts prenatally, whose regulatory mechanisms partially overlap with fetal fat accretion. Thus, colostrum content may undergo modifications to compensate for the harm of subtle TH deficiencies on neonates’ thermoregulation and development. Considering the previous findings showing that milk ω9 FAs are highest in colostrum, and even higher when mothers deliver preterm, our current results indicate their possible protective functions.

## 1. Introduction

A newborn’s consumption of their mother’s milk is essential not only for their early days but also for the succeeding developmental stages, supporting the fact that the mother’s milk profoundly influences babies’ health [1]. Breast milk is defined according to the lactation period, where colostrum, transitional milk, and mature milk approximately correspond to the first 6 days, 7 to 14 days, and after 15 days since delivery, respectively. Predictably, colostrum contains the most vital ingredients to protect neonates at their most vulnerable period after separating from the womb. Mothers’ colostrum and milk provide versatile benefits for the development of many organs, particularly neural and immune tissues, thanks to their bioactive compounds, including fatty acids (FAs). Respiratory tract diseases and mortality rates are lower in breastfed babies, and milk and colostrum components have neuroprotective and even anticancer properties [2,3]. Saturated and monounsaturated FAs are more abundant than polyunsaturated FAs in milk, and among the monounsaturated FAs, the most abundant is ω9 oleic acid, especially in the colostrum [4,5].

The human brain contains the highest lipid levels in the body after adipose tissue, where neurons utilize FAs with 18 carbons and oligodendroglia generate myelin, mainly based on lignoceric and ω9 nervonic acid with 24 carbons. Myelination starts at around the 32nd gestational week and continues considerably until the first 2 years of life [6,7]. In parallel, the accretion of saturated and ω9 FAs in the human brain is proportional to fetal brain weight during the last trimester [6]. Conceivably, the metabolic priming of breast epithelia starts before birth to produce milk in sufficient qualities for the newborn, and hence, the mothers’ metabolism affects the milk composition [8]. The mothers’ metabolic derangements may detrimentally affect the milk quality. Still, simultaneously, evolutionary mechanisms in mammalians would alter the cellular physiology of breast epithelia and colostrum components to lower the harmful influence of these conditions on newborns. For instance, nutrient restriction of beef heifers during late gestation reduces colostrum lactose but not its total fat and protein content, nor does it lower calf birth weight, since the maternal metabolism shifts to a catabolic state to partition nutrients for fetal growth and colostrum production [9].

Interestingly, the first two weeks after birth, where low thyroxine (T4) levels would permanently retard neonatal neurocognitive development, matches the production interval of colostrum and transitional milk [10]. Congenital hypothyroidism (CH), the most frequent cause of avertable intellectual disability, may occur due to a mother’s thyroid disorders, advanced maternal age, perinatal and labor complications, medications, and a newborn’s thyroid tissue abnormalities or prematurity [10]. CH is primarily asymptomatic and detected by evaluating blood levels of thyroid stimulating hormone (TSH), free thyroxine (FT4), or both [11]. Studies analyzing the correlations between milk composition and parameters such as birth weight are meager despite the mechanisms regulating milk content, and fetal fat accretion may partially overlap [8]. Although thyroid hormones (THs) profoundly affect the newborn’s metabolism and the physiology of breast epithelia, research on the ternary associations between TH, milk FA content, and birth weight is even more sparse. Thus, this study aimed to reveal the existence of these correlations and define the levels of 22 colostrum FAs. FAs’ percentage values were considered to assess their associations with birth weight to avoid the blurring effect of mothers producing different amounts of total colostrum fat. After revealing that ω9 monounsaturated and saturated FAs of over 18 carbon chain length correlate with birth weight in the whole cohort, further analyses focused on these regarding their associations as described above.

## 2. Materials and Methods

### 2.1. Ethics, Inclusion and Exclusion Criteria

Ethical approvals for this study were obtained from Acibadem University with approval numbers ATADEK-2013-507 and ATADEK-2023-16/565, respectively, where the former was related to colostrum and blood specimen collection and the latter to FA analysis, with the final date of ethical approval being 19th October of 2023. For the research grant application, the authors submitted their project to the Scientific Research Projects Coordination Unit (ABAPKO) of Acibadem University after obtaining the approvals mentioned above. The study was started following ABAPKO’s approval to finance the project on the 3rd of November, 2023. Informed consent forms were obtained from mothers who agreed to participate. All mothers were questioned whether they were diagnosed with any chronic diseases (i.e., hypertension, thyroid, or autoimmune disorders) or used medications, including iodine, amiodarone, lithium, carbamazepine, phenytoin, glucocorticoids, propranolol, and dopamine. The exclusion criteria were that the mothers had one of the above-cited health conditions, used the above-cited medicines, or developed diabetes or eclampsia during pregnancy, and whether the newborns were born with pre- or postmaturity. The inclusion criteria were mothers having singleton pregnancies and being able to provide sufficient amounts of colostrum (∼5 mL). Following these criteria, colostrum and blood samples from 78 mothers, aged 18 to 42, and their 78 newborns were analyzed.

### 2.2. Sampling and Analysis of Blood and Blood Spots

Blood samples from mothers were collected in the first 48 postpartum hours for analyzing serum levels of FT3, FT4, and TSH, where they were measured by electrochemiluminescence immunoassay (ECLIA) utilizing Elecsys 2010 (Roche Diagnostics, Mannheim, Germany). Newborns’ TSH was measured in heel-pricked dried blood spots after delivery, a routine procedure in Turkey for CH screening. Newborns’ TSH levels were defined by an enzymatic immunoassay (Neonatal TSH EIA Kit, TRIMARIS, Istanbul, Turkey).

### 2.3. Sampling and LC/MS-MS Analysis of Colostrum

Colostrum specimens were obtained within 48 postpartum hours from mothers who underwent vaginal delivery and within 72 postpartum hours from mothers who underwent C-sections via manual expression into sterile collection cups saved at −80 °C until analyses. For the FA analysis, all samples stored at −80 °C were brought to room temperature for thawing within the same period. A 2 mL sample of milk was mixed with 2 mL of ammonium hydroxide solution, 1 mL of ethanol, and 50 mg of pyrogallic acid (internal standard) and then hydrolyzed in a water bath at 70 °C for 20 min. Then, 4 mL of n-hexane/isopropanol (V/V = 3/2) solution was added to the hydrolysate, followed by oscillation and centrifugation to remove milk fat. Thereafter, 2.4 mL of n-hexane was used for additional extraction. The n-hexane extract was collected and mixed with 2 mL of methanolic NaOH (2%), capped, and heated at 50 °C in a water bath for 20 min for base-catalyzed methylation, followed by additional acid-catalyzed methylation processes using 2 mL of acetyl chloride–methanol (10%). After cooling to room temperature, 5 mL of n-hexane and 5 mL of ultrapure water were added to clarify the interface of the two phases. The extracted n-hexane was diluted and then analyzed by GC-MS. The FA composition was analyzed with a Thermo Scientific ISQ Trace 1300 Single Quadrupole GC-MS System (Thermo Scientific, Waltham, MA, USA) and a capillary column CP-Sil 88 (100 m × 0.25 mm × 0.25 µm). The injection volume was 1 μL, and the split ratio was 5:1. Helium was the carrier gas, and the Supelco 37 Component FAME Mix (Merc Company CRM47885, Darmstadt, Germany) was the standard sample to quantify FA levels.

### 2.4. Statistics and Normality Values

All statistical analyses were performed employing the Python programming language. Correlation coefficients and *p* values of coefficients were determined by the Spearman test. Heatmaps were created using Python’s Seaborn library. To examine the differences in median FA levels according to birth weight, mothers’ BMI, and FT4, the data distribution was first analyzed with the Kolmogorov–Smirnov test. After determining that the data were not normally distributed, a nonparametric Mann–Whitney U test was applied for comparisons. The statistical significance value was set as *p* < 0.05. After performing the statistics to define FA correlations with birth weight in the whole cohort, the data was stratified into subsets according to the mothers’ BMI, TSH, and FT4 values, as well as the newborns’ TSH. The analyses showed that only five of the 22 FAs correlated with birth weight in the whole cohort, comprising three ω9 monounsaturated FAs and two saturated FAs that can be produced with the same elongase enzymes. Therefore, further subgroup analyses were made these FAs having 18 to 24 carbons. In compliance with the standards of the World Health Organization (WHO), criteria to define mothers’ body mass index (BMI, mg/m^2^) as lower than normal, normal, overweight, and obese were <18.5, 18.5 to less than 25, 25 to less than 30, and 30 and over, respectively. According to these criteria, none of the mothers in the current cohort had lower-than-normal BMI. In compliance with the WHO standards, birth weight criteria of lower than normal, normal, and higher than normal were <2500 g, 2500 to less than 4000 g, and 4000 g and over, respectively. The normal ranges of thyroid hormones were defined according to the kits utilized for their measurements. The normal range of the mothers’ TSH were 0.27 to 4.2 mIU/L, and the normal ranges of the mothers’ FT3 and FT4 were 3.1 to 6.8 pmol/L and 12 to 22 pmol/L, respectively. Regarding the newborns’ TSH, values equal to or lower than 10 μIU/L are considered normal, while values over 10 μIU/L are regarded as high.

## 3. Results

Table 1 lists anthropometric and thyroid metabolism-related parameters of newborns and their mothers. The colostrum sample cohort comprised 78 women with a median age of 27 (18–42). The definitions used to describe subgroups stratified according to the anthropometric and thyroid hormone parameters, with their reference ranges given in the methodology, were as follows: normal, overweight, and obese BMI were defined for 15, 37, and 26 mothers, corresponding to 19.2%, 47.5%, and 33.3% of the entire cohort, respectively. There were 67 and 11 mothers with normal or high TSH, corresponding to 85.9% and 11.4% of the cohort, respectively. The study comprised 44 and 34 mothers with FT4 levels within the normal or higher-than-normal ranges, corresponding to 56.4% and 43.6% of the whole group. No mothers had TSH and FT3 values at lower-than-normal ranges or FT4 values at a higher-than-normal range. Numbers and percentages of newborns defined as those with normal or high TSH were 72 (92.3%) and 6 (7.7%), respectively. Lastly, 5, 67, and 6 newborns were described as having low, normal, or high birth weight, which corresponded to percentages of 6.4%, 85.9%, and 7.7% regarding the whole cohort. The findings regarding FT3 levels are neither addressed here nor in the discussion, since a meager number of mothers had high FT3 levels, precluding appropriate statistical analyses. Also, all *p* and r values are not given in this section to avoid giving redundant information.

Table 2 shows total colostrum FA μg/mL levels, stratified according to newborns’ birth weight and TSH, mothers’ TSH, FT4, and BMI values, and ω9 and saturated FA amounts in the entire cohort. Total FA µg/mL levels showed a high divergence, with a mean value of 895.2 µg/mL ranging between 13.3 and 3011.3 µg/mL. Percentages of ω9 gondoic and erucic acids and those of saturated arachidic acid correlated with the total FA amount in the entire cohort. These correlations were always positive, with *p* values of 0.002, 0.002, and 0.010, respectively. When all 22 FAs in colostrum were compared between the subgroups consisting of newborns with lower-than-normal, normal, and higher-than-normal birth weights, stearic acid was found to be significantly (*p* = 0.022) higher in the subgroup of newborns with lower-than-normal birth weight than normal. In contrast, gondoic acid in colostrum was significantly (*p* = 0.007) higher in the subgroup of newborns with higher-than-normal birth weight than normal. Pairwise comparisons of the total colostrum fat level for groups separated according to newborns’ birth weight and TSH, and mothers’ TSH, FT4, and BMI values did not reveal any statistically significant difference. ω9 oleic and saturated stearic acids, both having 18 carbons, were the most abundant FAs.

Considering the standard deviation values, there was a tendency of declining variability proportional to lesser carbon lengths of FAs, where ω9 oleic and saturated stearic acids showed the lowest, while ω9 nervonic and saturated lignoceric acids showed the highest divergence. In decreasing order, ω9 gondoic, erucic, and nervonic acids existed at percentages much lower than oleic acid. Still, their levels at μg/mL were within and occasionally above the necessary amounts for the biological activity, as considered in the discussion. Except for the most abundant stearic acid, saturated FAs’ percentage and μg/mL amounts increased proportionally to their carbon length in the order of arachidic < behenic < lignoceric acid.

**Figure 1:**
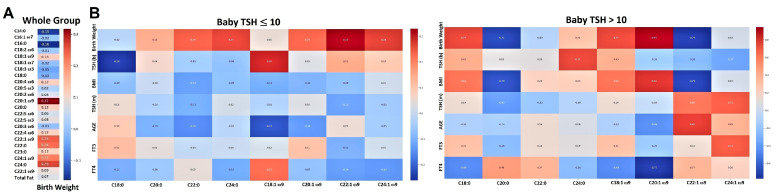
Correlations of the colostrum’s total fat and percentages of 22 FAs and with birth weight in the whole cohort (**A**). Correlations of ω9 and saturated FA percentages with birth weight stratified according to the range values of newborns’ TSH (**B**).

Figure 1A demonstrates the correlations of birth weight with total FA amount and percentages of ω9 monounsaturated and saturated FAs relevant to the whole cohort. Figure 1B depicts the same correlations in subgroups stratified according to newborns’ TSH.

**Figure 2:**
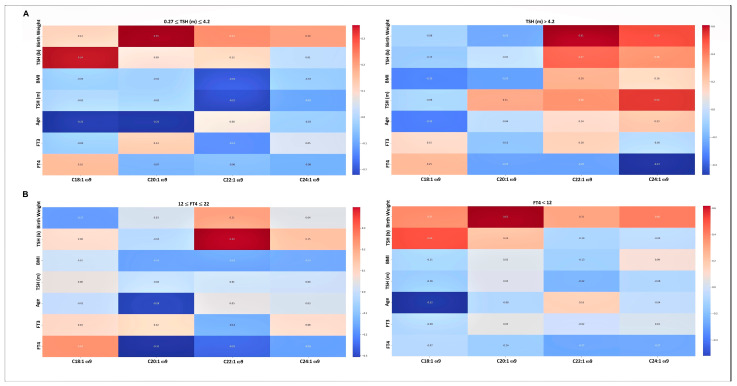
Correlations of ω9 FA percentages with birth weight stratified according to the range values of mothers’ TSH (**A**) or FT4 (**B**).

Figure 2 depicts correlations of ω9 FAs with birth weight, mothers’ and newborns’ TSH, and mothers’ FT3, FT4, and age values, where Figure 2A,B represents subgroups separated according to mothers’ TSH and FT4 levels, respectively.

Table 3 lists the correlation coefficiency and *p* values of correlations of ω9 and saturated FAs with birth weight, newborn’s TSH, and mother’s age, all of which were analyzed in the whole cohort and separated according to the ranges of mother’s BMI, TSH, and FT4, and the ranges of newborns’ TSH. Supplementary Table S1 presents the same data after excluding insignificant results, and for each FA, correlations with birth weight, newborns’ TSH, and mothers’ age were summarized in the same line to provide a compiled view and ease the tracking of main information.

Oleic acid, which did not correlate with birth weight in the whole cohort, positively correlated with birth weight in the subgroup, including mothers with low FT4. Oleic acid correlated positively with newborns’ TSH in the whole cohort and among mothers with obesity, normal TSH, or low FT4 values, and among newborns having normal TSH (*p* values equal to 0.021, 0.010, 0.005, 0.003, and 0.009, respectively). Oleic acid negatively correlated with mothers’ age in the entire cohort and among mothers with low FT4 values or obesity. Gondoic acid positively correlated with birth weight in the whole cohort and among mothers with obesity, normal TSH, or low FT4, and newborns with normal or high TSH. These correlations had the highest significance in groups consisting of mothers with low FT4 or newborns with high TSH. Erucic acid positively correlated with birth weight in the general cohort and subsets, including mothers with normal BMI or high TSH levels and newborns with normal TSH levels (*p* values equal to 0.030, 0.005, 0.005, 0.047, and 0.005, respectively). Erucic negatively or positively correlated with newborns’ TSH in groups encompassing mothers with obesity or having normal FT4 levels, respectively, with a higher significance for the latter association. Erucic acid positively correlated with the mothers’ age only in the subset comprehending newborns having high TSH. Nervonic acid correlated positively with birth weight in the whole group and among mothers with low FT4 or newborns with normal TSH (*p* values equal to 0.049, 0.019, and 0.018, respectively). Nervonic acid also exerted a positive correlation with mothers’ age among obese mothers. Stearic acid negatively correlated with birth weight among mothers with high TSH levels. Stearic acid also negatively correlated with the newborn’s’ TSH in the whole cohort and among newborns having normal TSH values (*p* values equal to 0.036 and 0.027, respectively). Arachidic acid did not correlate with any parameter except for a positive correlation with the newborns’ TSH among mothers having normal BMI. Behenic acid was positively associated with birth weight in the whole cohort and among mothers with normal BMI or TSH values or low FT4 levels (*p* values equal to 0.036, 0.010, 0.016, and 0.002, respectively). This FA also positively correlated with birth weight among newborns having normal TSH levels. Lignoceric acid’s correlations exerted patterns the same as behenic acid; neither behenic and lignoceric acids nor any saturated long-chain FAs correlated with the mothers’ age in any of the evaluated groups.

## 4. Discussion

### 4.1. Fetal Fat Accrual, Birth Weight, and Colostrum Protection Against Hypothermia

Globally, low birth weight and hypothermia cause a high percentage of neonatal death since lower birth weight increases the surface area-to-volume ratio and heat loss in newborns [12]. Among all mammals, the highest fetal fat expansion occurs in humans to protect neonates against a rapid temperature shift from intrauterine to post-delivery life [13]. Carbohydrates and lipids comprise newborns’ primary heat production substrates. The latter determines the time the carbohydrates can last, and inadequate liver glycogen in preterm infants increases the dependence on fat stores [14]. In mammalians, evolutionary mechanisms exist to minimize the effects of mothers’ excess weight or insufficient nutrition on the fetus. For instance, mothers’ obesity or fasting did not significantly influence fetal weight gain in piglets, while the latter increased nonsterified FAs in the mothers’ blood, induced adipocyte hyperplasia, and decreased lipoprotein lipase activity in fetal adipose tissue to compensate for the harmful effects of calorigenic decline [15]. Fetal lipogenesis provides a robust fat gain starting from the 30th gestational week, and lipogenic enzyme activities close to birth and in newborns are more prominent than in adults, with preterm neonates being small at gestational age to synthesize FAs at even higher rates [13]. In 1992, Ohno and colleagues published that the weight, phospholipids, polyunsaturated FAs, arachidonic indices in triglycerides, and eicosadienoic and lignoceric acid percentages are higher in the brown adipose tissue of pre-weaning rats than those of cold-acclimated adults [16]. This study provided one of the early hints that neonatal brown adipose tissue features resemble those encountered in adult organisms during cold adaptation and helped to establish connections between neonatal brown adipose tissue and metabolic defenses against hypothermia. For cold acclimation, colostrum alkylglycerols activate newborns’ brown fat, which has high levels of mitochondrial uncoupling protein [UCP] that converts the metabolic energetic sources into heat, whose activity is highest in neonatal life [16]. The following sections discuss the associations between brown fat activation, FAs, and TH after explicating the possible causes of encountering a relatively high percentage of subtle hypothyroid status in the cohort.

### 4.2. Putative Reasons for Encountering Hypothyroid Stage at a High Percentage

No universally accepted reference ranges exist for TSH to diagnose CH since some countries prefer stricter criteria at the expense of making excrescent tests, as excess testing is more acceptable than overlooking CH causing permanent neurocognitive deficits [10]. Iodine-based skin disinfectants are commonly used in Turkey [11]. This fact and the relatively low upper range of TSH (10 μIU/L) employed in this study may have caused six (7.7%) newborns to encounter possibly transient hypothyroidism. Despite 11 (14.1%) mothers having TSH values above normal, 34 (43.6%) mothers had subphysiological FT4 levels, which may be because FT4 reductions were not prominent enough to stimulate TSH secretion. Here, the weight factor may have been dominant since 80.8% of mothers in this cohort had BMI values above the normal range. TSH positively correlates with BMI as it decreases through negative feedback from TH, which accelerates basal metabolism, and the gestational BMI of mothers inversely correlates with their FT4 levels. The influence of BMI increases due to the altered deiodinase activity and expanding adipose tissue with progressing pregnancy, with enhanced iodine demand for fetal thyroid tissue support and increases in TSH, FT3, FT3/FT4 ratio even among euthyroid pregnant [17,18,19]. The iodine factor may be significant for Turkey, as a study published in 2021 stated that 19% of Turkish lactating mothers are hypothyroid, attributable to iodine deficiency [20]. Newborns of mothers weighing higher than normal tend to be large for gestational age, explaining that 7.7% of newborns have a higher-than-normal birth weight in this cohort. Still, this percentage is much lower than that of mothers who weigh more than normal, as mothers’ weight has a limited influence on fetal weight gain [15]. Here, one could propose that ω9 and saturated VLCFAs’ positive correlations with birth weight may be due to hypothyroidism harmfully affecting mothers and, subsequently, newborns’ weight. Yet, except gondoic acid, none of the ω9 and saturated VLCFAs correlated with birth weight among mothers with BMI values over normal. Furthermore, total colostrum fat did not differ between subgroups separated according to mothers’ BMI or newborns’ birth weight, excluding this possibility.

### 4.3. Thyroid Hormones Regulating Fetal Adipogenesis, Brown Fat and Colostrum

Thyroid hormones regulate lipid metabolism through mobilizing adipose tissue triglycerides, increasing hepatic lipase activity, and lipid utilization [16]. In humans, mothers’ blood lipids increase gradually to meet fetal metabolic demands, which occurs at higher rates during subclinical hypothyroidism [16]. Although TH acceleration of basal metabolism and weight loss in adults is well established, the effects of mothers’ hypothyroidism on fetuses are less known. Treatment of pregnant rats with an antithyroid agent leads to offsprings’ lesser birth weight, concomitant with reductions in lipogenesis and expression of genes regulating FA uptake [21]. TSH has lipolytic effects beyond affecting thyroid tissue through its receptor in several cells, including adipocytes and hepatocytes, where it stimulates lipolysis [16]. While mothers’ hypothyroidism may harm neonates, mothers’ high TSH-driven lipolysis, subsequently increasing FAs, and colostrum FA modifications may provide compensatory protections. In this study, oleic acid positively correlated with birth weight only among mothers having low FT4 levels, which is the same subgroup where gondoic, behenic, and lignoceric acid correlations with birth weight had the highest significance. In the same group, erucic acid showed a tendency for a positive correlation with birth weight (*p* = 0.075), which reached statistical significance among mothers having high TSH values. This phenomenon may be associated with a lack of transplacental passage of TSH, while T4 can do so. Hence, metabolic crosstalk between mothers and developing fetuses may depend more on T4, causing colostrum content adjustments based more on T4 levels.

TSH upregulates deiodinase enzymes in brown fat to convert the circulating T4 into T3, which increases acylcarnitines and stimulates FA β-oxidation for thermogenesis [22,23]. Therefore, thyroid hormones are paramount in the newborn’s cold adaptation, and newborns with low birth weight have higher TSH, presumably as an adaptation [24]. Inadequate TH status may also be compensated through nutrients. Indeed, a high-fat diet after birth elevates T4 levels in lambs, and colostrum intake positively correlates with blood TH levels and higher pre-weaning growth in suckling piglets [25,26]. Feeding dairy cattle with thyroprotein having thyroxine-like activity reduces colostrum FAs with carbon chain lengths of 12, 14, and 16 and increases FAs with carbon lengths 18, raising oleic acid levels by 80% [27]. In parallel, T3 injection in rats induces a sharp elevation in stearoyl-coA desaturase (SCD)/Δ9 desaturase activity that converts stearic to oleic acid [28]. These findings concur with the observations regarding stearic acid, including the following: it is higher in the colostrum of mothers delivering newborns with lower-than-normal birth weight, correlates negatively with birth weight among mothers with high TSH, and correlates negatively with newborns’ TSH in the whole cohort and among newborns with normal TSH. All these features may stem from inadequate TH levels enhancing stearic acid levels by diminishing its elongation and causing the delivery of low-birth-weight babies. This proposal makes sense considering the above-declared parallelities between fetal adipogenesis and colostrum FA regulation and also the fact that stearic acid is the most abundant LC-SFA in colostrum, which, in this cohort, existed at levels 16-fold higher than the second-most abundant LC-SFA, lignoceric acid. Stearic acid’s negative correlation with newborns’ TSH may be secondary to indistinct tenuous delays of intrauterine development, including hypothalamic maturation. Oleic acid was the single ω9 FA that did not correlate with birth weight in the general cohort despite the other ω9 FAs showing this correlation are produced with oleic acid elongation. This paradox may be associated with oleic acid existence at much higher levels than other ω9 FAs, hindering the finding of a statistical association. Compensatory modifications of less abundant FAs would have a higher influence on percentages, leading to increased statistical differences, which are produced with the same enzymes described below.

### 4.4. Same FA Elongase Enzymes Produce ω9 and Saturated VLCFAs

**Figure 3:**
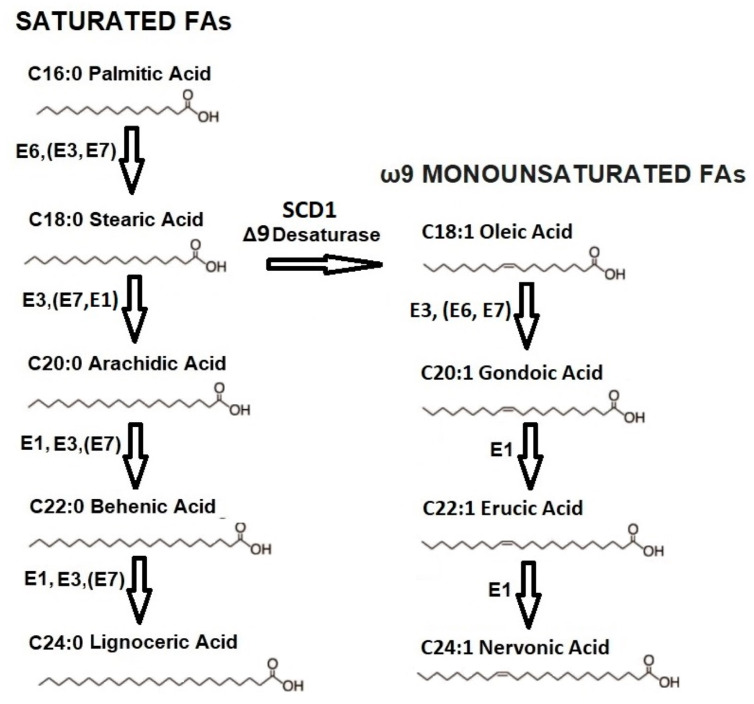
Elongation of ω9 and saturated FAs catalyzed by ELOVL enzymes (E1, E3, E6, E7) and Δ9 desaturation of stearic acid to oleic acid catalyzed by stearoyl-coA desaturase 1 (SCD1). ELOVLs (i.e., E1, E3) written before the parentheses represent the main enzyme catalyzing the elongation, and those written inside the parentheses represent their stablemates, which redundantly exert the same function.

Fatty acid synthase (FAS) enzymes produce FAs with chain lengths from two to sixteen carbons [7]. ELOVL (Elongation of VLCFA) enzymes are encoded by seven genes in humans, whose expressions undergo changes in diet, hormones, and developmental stages and catalyze the synthesis of FAs with chain lengths of16 and above by adding a two-carbon unit at each step. ELOVL1, ELOVL3, and ELOVL6 could only elongate saturated and monounsaturated FAs, including ω9 FAs depicted in Figure 3.

Elongases show mutual redundancy, where ELOVL1 is redundant with ELOVL3 and ELOVL7 and ELOVL3 is redundant with ELOVL1 and ELOVL7. VLCFAs participate in complex lipid structures like the brain phospholipids and ether phospholipids, which form skin barriers [7]. ELOVL3 and ELOVL6 are expressed in adipose tissue, skin, and liver, and their deficiencies perturb dermal lipids and insulin metabolism [29]. ELOVL3 is highly induced in brown fat for cold acclimation, and ELOVL3 knock-out animals have reduced adipose mass and impaired insulation [30]. However, some studies showed C20 and C22 VLCFA accumulation in ELOVL3 knock-out mice, which may be due to the redundant features of ELOVLs, where the loss of one may be compensated with a robust upregulation of another [31]. The fact that there is a paucity of data about the expression and compensatory regulation of ELOVL enzymes outside the rodents’ breast epithelia indicates the requirement for future human studies on these issues. Mammalian cells also express desaturase enzymes that introduce C=C double bonds in the FA’s backbone at the 5th, 6th, and 9th positions, therefore named Δ5, Δ6, and Δ9 desaturases, respectively, and Δ9 desaturases producing ω9 FAs are also designated SCDs. Humans express SCD1 ubiquitously, and SCD1 knock-out mice are severely depleted in triacylglycerols and skin lipids and are highly cold-intolerant [30]. The succeeding section describes similar results about colostrum ω9 FAs obtained in different geographies.

### 4.5. ω9 FAs Exist the Most in Colostrum. Parallelities Between Different Geographies

The positive correlation of colostrum’s total FA content with ω9 gondoic and nervonic acids, as well as the fact that 3 of the 5 FAs positively correlating with birth were ω9 FAs, may establish a link to their putative benefits. Since ω9 FAs are endogenously synthesized, they have been sparsely studied, which also applies to colostrum research mainly reported from Asian countries [32,33,34,35]. Strikingly, both Asian and European studies have found that levels of oleic acid and, most of the time, other ω9 FA levels are highest in colostrum and decrease as lactation progresses [4,33,36,37,38,39]. A Hungarian study showed that ω9 gondoic, erucic, and nervonic acids are higher in colostrum than in transitional and mature milk and that these levels are even higher in samples of mothers who gave births prematurely [40]. A Polish study found that erucic acid levels are higher in colostrum than in mature milk [41]. The same results obtained in different geographies indicate benefits of ω9 FAs for the newborn, an assumption supported with recent studies. Infant growth negatively correlates with medium-chain saturated FAs but positively with oleic acid [42]. It is well established that vaginal delivery is more beneficial for mothers and newborns than cesarean delivery, and oleic and erucic acid levels in the milk of mothers who gave birth vaginally are higher than those who gave birth by cesarean [43]. Below, ω9 FAs will be discussed regarding their roles in fetal fat gain and neonates’ physiology.

### 4.6. ω9 FAs in Neonatal Thermogenesis and Development

As mentioned, oleic acid positively correlated with birth weight only in the subgroup encompassing mothers with low FT4. Oleic acid positively correlated with newborn TSH, mainly among mothers and newborns with normal TSH. The same FA negatively correlated with mothers’ age in the entire cohort and among mothers with obesity or low FT4. A collective evaluation of these correlations provokes the idea that oleic acid production and release into colostrum require proper TH levels, restricting its compensatory increase under hypothyroid conditions due to mothers’ obesity or relatively higher age. Despite not correlating with birth weight in the general cohort, oleic acid is one of the primary FAs in brown fat and in thermogenic stimulation by increasing UCP and activating the pivotal brown adipocyte receptor GPR3 [28,29,30]. Therefore, newborns with even elusive TH deficits may benefit from oleic acid supplementation, as its synthesis may depend on adequate TH levels. Oleic acid may benefit newborns through additional mechanisms, such as protecting mitochondria and providing better blood lipid profiles and antioxidant capacity, as revealed by a meta-analysis of 67 human clinical trials [31,32]. In colostrum, oleic acid is particularly complexed with phospholipids and contributes to the phospholipid-rich brain development, where the newborn brains could uptake it from the blood to support synaptogenic growth cones, while feeding newborn mice with its amide complex robustly enhances cognitive test scores [4,38,40,44,45].

As declared, gondoic acid was more abundant in the colostrum of mothers delivering higher-than-normal birth weight babies. The same FA positively correlated considerably significantly with birth weight in the entire cohort and showed the exact correlation among mothers with obesity, normal TSH, or low FT4 levels and among newborns having normal or high TSH values. Gondoic acid’s positive correlation with birth weight among mothers with low FT4 levels had the highest significance out of all the associations in this study. This feature may be associated with the chain elongation of gondoic acid from oleic acid involving ELOVL3, ELOVL6, or ELOVL7, while the elongation of ω9 FAs with more than 20 carbons is catalyzed only by ELOVL1. Here, there exists the likelihood that ELOVL1 elongation of ω9 FAs may not be stimulated when mothers have a BMI above normal. Meager studies about gondoic acid showed opposite protective and detrimental effects. Yet, it is unlikely that colostrum levels of gondoic acid act harmful to the newborn. Gondoic acid prevents lipotoxicity in pancreatic β cells secreting insulin, synergizes with ω3 FAs to reduce excess fat accumulation in hepatocytes, and its levels in serum phospholipids of premenopausal women negatively correlate with BMI [46,47,48,49]. Gondoic acid may also support neurodevelopment. Quaking mutant mice for the qkI gene and those suffering from tremors have drastically decreased myelin and its gondoic, erucic, and nervonic acid content, with gondoic acid derivatives potently inhibiting acetylcholine esterase that degrades acetylcholine, the primary learning and memory neurotransmitter [50].

Erucic acid correlated positively with birth weight among mothers with normal BMI or newborns with normal TSH. This FA also correlated positively with the mothers’ age among newborns with high TSH. Lastly, erucic acid negatively correlated with newborns’ TSH among obese mothers. These correlations may be interconnected and raise the idea that erucic acid synthesis requires adequate TH levels similar to oleic acid. In this study, erucic acid was measurable in all samples, likely due to both endogenous synthesis and erucic acid-rich vegetable consumption in Turkey, similar to those in Far Asia [31,51,52]. The corresponding author of this study and colleagues were the first to publish that erucic acid could inhibit glioblastoma cell growth and increase the delivery of anticancer plasmid DNA, publishing reviews that comprised analyses about this FA in health and disease [6,51,52]. As revealed from these studies, erucic acid has been considered mainly toxic due to deaths in persons consuming rapeseed oil containing this FA. Nonetheless, it was later found that these deaths were related only to consuming oils refined by toxic anilines for industrial use, and toxicity in humans did not occur when erucic acid was used to treat adrenoleukodystrophy (ALD) [51,52].

Lipoprotein lipase (LPL), which provides FAs through triglyceride hydrolysis, may be responsible for the positive correlation of erucic acid with birth weight, since feeding rats with this FA enhances LPL activity in the heart, liver, and adipose tissue, the latter of which does not involve and even reducing hormone-stimulated activity [53]. This feature was attributed to the reversal of cardiac lipidosis in rats after an initial period of erucic acid feeding. Peculiarly, feeding pregnant rats even with high-dose erucic acid does not cause lipidosis, suggesting this FA may be a safe source of fetal adipogenesis [54]. Colostrum erucic acid levels were very variable, reaching up to 430.3 μM, while erucic acid’s therapeutic blood concentrations in ALD start from 125 μM [6]. As ALD is a pediatric demyelinating disease, erucic acid would benefit myelination and reduce pediatric brain tumors since it activates transcription factor PPARδ, mediating neuroglial differentiation [52]. Indeed, in preclinical studies, erucic acid lowers glioblastoma cell growth more than benign mesenchymal cells and improves cognitive functions [6,52]. Lastly, erucic acid suppresses the pregnancy aggravation of systemic lupus erythematosus and enhances intestinal colonization of the probiotic bacteria, *Lactobacillus johnsonii* [55,56]. Therefore, erucic acid may reduce newborn deaths by preventing autoimmune diseases and necrotizing enterocolitis.

In addition to the sparsity of research about ω9 FAs’ role in human health, evidence regarding erucic and gondoic acid health benefits are mainly obtained from animal studies, a fact even more prominent for gondoic acid. Therefore, it would be improper to extrapolate animal study results directly to human health. Several study types would contribute to improving the knowledge about these FAs, such as analyzing their blood level correlations with human diseases and employing experiments in transgenic rodents, which are “humanized” according to several components of those related to immunology, neurobiology, and others.

Nervonic acid’s positive correlations with birth weight among mothers with low FT4 and with obese mothers’ age also recall its compensatory regulation against inadequate TH levels. Nervonic acid induces mesenchymal stem cell differentiation into adipocytes while limiting diet-induced weight gain [57,58]. These two effects are not contradictory; instead, they indicate the likelihood of nervonic acid simultaneously accelerating fetal fat accrual and utilizing FAs from brown fat, both protective against hypothermia. Supporting this presumption, nervonic acid in human epicardial fat correlates positively with adiponectin regulating FA oxidation, with lower levels associated with obesity and higher levels with reductions in diabetes risk [59]. Nervonic acid was about two times lower than erucic acid in colostrum, possibly due to the insufficient elongation of its precursor ω9 FAs in the periphery. Another factor may be its existence limited mainly to medicinal plants such as Lunaria, Malania, and Acer truncatum, which are used for demyelinating diseases in Asian medicine. Nervonic acid is a component of myelin sphingolipids, and it rapidly accumulates in the fetal brain during the last trimester [60,61,62]. Some could presume that nervonic acid’s low levels in colostrum would preclude bioactivity, but animal studies have proved the opposite. Feeding pregnant rats with canola oil, containing nervonic acid at much lower amounts than the medicinal plants, did not change fetal tissue nervonic acid levels. Still, feeding lactating rats with the same oil increased nervonic acid in suckling rats’ hearts and livers [63]. These findings could be relevant to humans due to the following observations: Head circumference in preterm infants and better postnatal neuromental development in term infants correlate with higher nervonic acid levels in the placenta and cord blood, respectively; and blood nervonic acid levels of mothers who gave birth to healthy babies are higher than those who delivered babies with neural tube defects [64,65,66]. Lastly, nervonic acid could reduce necrotizing colitis risk in infants since it inhibits intestinal injury and macrophage release of inflammatory mediators after bacterial lipopolysaccharide stimulation [67].

### 4.7. Long-Chain Saturated FAs in Neonatal Thermogenesis and Development

Behenic and lignoceric acids, positively correlating with birth weight with patterns almost identical to ω9 FAs, also possess physiological functions very similar to ω9 FAs. Arachidic and behenic acids have principal roles in brown fat triglyceride synthesis, which are depleted in ELOVL3 knock-out mice intolerant to cold [68]. A study analyzing 29 human milk FAs revealed that behenic acid is one of the only two FAs that positively correlated with sphingomyelin and phosphatidylcholine levels, which are crucial for neural and intestinal maturation [5]. In a gestational diabetes model, behenic acid feeding prevents glucose and insulin metabolism defects, abnormal weight gain, and offspring mortality [69]. Lignoceric acid correlations with mothers’ TSH and FT4 levels positively and negatively, respectively, may be associated with the inadequate TH levels hampering ELOVL1 activity. Lignoceric acid exists at the highest levels in colostrum and gradually declines with progressing lactation [70]. In hibernating animals, lignoceric acid is utilized in brown fat for cold acclimation, concurrent with its high-level presence in pre-weaning rats’ brown fat, which declines after weaning [16,71]. In adult humans, cold exposure increases the blood’s lignoceric but decreases behenic, eicosanoic, and nervonic acids [72]. This feature may be attributed to a rapid entrance of thermogenic FAs from the blood to brown fat, when lignoceric acid may not enter the brown fat at the same pace after liberation.

Saturated VLCFAs in colostrum may support neonates with additional functions. In humans, behenic acid is lower in amniotic fluid sphingomyelin among diabetic pregnant mothers than in healthy ones, which is parallel to the importance of sphingomyelin in fetal neurodevelopment and its detrimental influence by diabetes [73]. Swine brain studies showed that behenic to lignoceric acid elongation occurred in neonatal brains but not adult ones, indicating a higher requirement for lignoceric acid during neurodevelopment [74]. In newborn mouse cerebellum cell cultures and rat brains, an enzyme α-hydroxylates nervonic and lignoceric acids, where the latter provides the myelin component cerebronic acid [75]. In rat brains, this enzyme activity rapidly increases after birth until the 23rd postnatal day, which is retarded by neonatal hypothyroidism and accelerated by TH, explained by the significant demand for TH in myelination [76]. This feature resembles the phenomenon where inadequate TH levels in a narrow period impair human neurodevelopment. Consistently, lignoceric acid in human brain cerebrosides increases rapidly towards the latest gestational periods, and human cord blood lignoceric and oleic acids positively correlate with newborns’ social interaction and development up to 18 months [58,65]. Collectively, ω9 and saturated VLCFAs may provide wide-ranging health benefits for neonates.

## 5. Limitations and Strengths

As reaching a sample size adequate to analyze the separate associations of three anthropometric values and three TH parameters that can provide information about different physiological values is challenging, the sample size is relatively small. Still, the results showed logical parallelities, indicating they merit further study. Applying strict inclusion and exclusion criteria and evaluating FA percentage levels specific to each sample to avoid blurring effects of mothers producing different amounts of total colostrum fat constitute the strengths of this study.

## 6. Conclusions

Considering the overlapping features of ω9 and saturated VLCFAs, including correlations with birth weight, akin to patterns related to TH status, production with the same enzymes, and roles in thermoregulation and development, it is conceivable to presume that their colostrum levels may undergo simultaneous modifications to support neonatal health. This assumption makes further sense considering that none of the essential FAs (three ω3 and six ω6), which are not produced endogenously and therefore not adjustable, correlated with birth weight. Several research strategies may be implemented to improve the knowledge of this almost completely unexplored scientific field. First, the results of this current study need to be repeated by further studies to obtain firm opinions. Second, studying the effects of thyroid hormones on the expression of elongase enzymes and FA synthesis in primary human breast epithelial cell cultures and xenogenic tissue implants would provide crucial data. Third, analyzing the effects of conceptional hypothyroidism on fetal adipogenesis, birth weight, neonatal volume, and activation of brown fat in humanized transgenic rodents would contribute essential information about quadruple associations of TH, brown fat, and the regulation of both fetal adipogenesis and colostrum FA synthesis. If upcoming studies support current findings and additional research is conducted in this novel field, these will help develop innovative nutritional strategies for mothers and newborns for common endocrine problems.

## Figures and Tables

**Table 1:** Demographic, anthropometric, and thyroid hormone values.

**Age**	**Mean ± STD**	27.42 ± 5.30			
	**Median (min–max)**	27 (18–42)			
		**Whole Cohort**	**Normal**	**Overweight**	**Obese**
**BMI**	*n* (%)	78 (100%)	15 (19.2%)	37 (47.5%)	26 (33.3%)
	Mean ± STD	28.6 ± 4.1	23.2 ± 1.3	27.4 ± 1.5	33.3 ± 2.3
	Median (min–max)	28.1 (20.9–37.7)	23.3 (20.9–24.9)	27.3 (25.0–29.9)	33.1 (30.0–37.7)
		**Whole Cohort**	**Low**	**Normal**	**High**
**Mother TSH**	*n* (%)	78 (100%)	0 (%)	67 (85.9%)	11 (14.1%)
	Mean ± STD	2.9 ± 2.4	-	2.2 ± 0.9	7.1 ± 4.2
	Median (min–max)	2.4 (0.6–19.2)	**-**	2.0 (0.6–4.2)	6.0 (4.5–19.2)
**Mother FT3**	*n* (%)	78 (100%)	0 (%)	76 (97.4%)	2 (2.6%)
	Mean ± STD	4.7 ± 0.8	-	4.7 ± 0.7	7.5 ± 1.0
	Median (min–max)	4.5 (3.4–8.2)	-	4.7 (3.4–6.6)	7.5 (6.9–8.2)
**Mother FT4**	*n* (%)	78 (100%)	34 (43.6%)	44 (56.4%)	0 (%)
	Mean ± STD	12.3 ± 2.1	10.4 ± 1.1	13.7 ± 1.5	-
	Median (min–max)	12.1 (7.9–18.0)	10.7 (7.9–11.9)	13.2 (12.0–18.0)	-
**Birth Weight**	*n* (%)	78 (100%)	5 (6.4%)	67 (85.9%)	6 (7.7%)
	Mean ± STD	3218.5 ± 495.2	2208.0 ± 247.3	3209.6 ± 351.5	4160.0 ± 100.9
	Median (min–max)	3160 (1780–4340)	2260 (1780–2400)	3160 (2600–4000)	4135 (4050–4340)
**Newborn TSH**	*n* (%)	78 (100%)	0 (%)	72 (92.3%)	6 (7.7%)
	Mean ± STD	4.3 ± 2.9	-	3.7 ± 2.2	11.5 ± 0.8
	Median (min–max)	3.8 (0.1–12.3)	-	3.5 (0.1–9.8)	11.5 (10.1–12.3)

**Table 2:** Levels of total fat, and ω9 monounsaturated and saturated FAs.

**Total Fat—Whole Cohort**				Median: 895.2 (13.3–3011.3) µg/mL			
**Total Fat—µg/mL (Mean ± STD)**							
**BMI—Normal**		**BMI—Overweight**				**BMI—Obese**	
669.8 ± 471.7 *		605.3± 533.4 *				524.8± 428.9 *	
**Birth Weight—Low**		**Birth Weight—Normal**				**Birth Weight—High**	
470.5 ± 318.6 *		599.0 ± 506.4 *				600.9 ± 390.6 *	
**TSH (newborn)—Normal**				**TSH (newborn)—High**			
593.7 ± 500.0 *				556.9 ± 286.7 *			
**TSH (mother)—Normal**				**TSH (mother)—High**			
586.5 ± 510.3 *				617.7 ± 312.1 *			
**FT4 (mother)—Normal**				**FT4 (mother)—Low**			
664.6 ± 564.4 *				495.5 ± 345.0 *			
**ω9 Monounsaturated FAs—Whole Cohort**							
	**Oleic Acid**		**Gondoic Acid**		**Erucic Acid**		**Nervonic Acid**
**Level (%)**	29.5 ± 2.3		0.67 ± 0.16		0.30 ± 0.13		0.15 ± 0.07
**Level (µg/mL)**	17,985.4 ± 15,101.7		435.9 ± 445.9		180.0 ± 176.0		101.4 ± 111.4
**Saturated FAs—Whole Cohort**							
	**Stearic Acid**		**Arachidic Acid**		**Behenic Acid**		**Lignoceric Acid**
**Level (%)**	7.2 ± 1.3		0.08 ± 0.05		0.31 ± 0.13		0.45 ± 0.62
**Level (µg/mL)**	4329.6 ± 3604.4		53.8 ± 56.7		177.0 ± 159.1		204.2 ± 194.1

* *p* > 0.05 for pairwise comparisons according to the Mann–Whitney U test.

**Table 3:** FA correlations with newborns’ birth weight and TSH and mothers’ age.

FAs		Whole Group	BMI Normal	BMI Overweight	BMI Obese	TSH Mother Normal	TSH Mother High	FT4 Mother Normal	FT4 Mother Low	TSH Newb. Normal	TSH Newb. High
ω9 FAs		Birth Weight	Birth Weight	Birth Weight	Birth Weight	Birth Weight	Birth Weight	Birth Weight	Birth Weight	Birth Weight	Birth Weight
**Oleic**	**r**	0.12	0.13	0.06	0.21	0.12	−0.08	−0.17	0.37	0.05	0.77
	***p***	*0.251*	*0.230*	*0.882*	*0.335*	*0.343*	*0.811*	*0.268*	***0.029***	*0.685*	*0.072*
**Gondoic**	**r**	0.32	0.40	0.16	0.45	0.35	−0.23	0.03	0.62	0.24	0.94
	***p***	***0.005***	*0.169*	*0.534*	***0.031***	***0.004***	*0.502*	*0.863*	***0.0001***	***0.046***	***0.005***
**Erucic**	**r**	0.25	0.63	0.26	−0.02	0.23	0.61	0.21	0.31	0.33	−0.77
	***p***	***0.030***	***0.005***	*0.155*	*0.923*	*0.067*	***0.047***	*0.163*	*0.075*	***0.005***	*0.072*
**Nervonic**	**r**	0.22	−0.13	0.28	0.26	0.20	0.50	0.04	0.40	0.28	0.03
	***p***	***0.049***	*0.545*	*0.438*	*0.285*	*0.105*	*0.117*	*0.791*	***0.019***	***0.018***	*0.957*
**ω9 FAs**		**TSH Newb.**	**TSH Newb.**	**TSH Newb.**	**TSH Newb.**	**TSH Newb.**	**TSH Newb.**	**TSH Newb.**	**TSH Newb.**	**TSH Newb.**	**TSH Newb.**
**Oleic**	**r**	0.26	0.12	0.16	0.48	0.34	−0.13	0.08	0.50	0.30	0.43
	***p***	***0.021***	*0.867*	*0.450*	***0.010***	***0.005***	*0.709*	*0.593*	***0.003***	***0.009***	*0.397*
**Gondoic**	**r**	0.12	−0.09	0.18	0.13	0.09	−0.02	−0.03	0.24	0.03	−0.20
	***p***	*0.298*	*0.542*	*0.480*	*0.349*	*0.452*	*0.947*	*0.163*	*0.866*	*0.808*	*0.704*
**Erucic**	**r**	0.02	0.04	0.18	−0.45	0.12	0.47	0.40	−0.10	0.15	−0.31
	***p***	*0.877*	*0.923*	*0.395*	***0.015***	*0.341*	*0.145*	***0.007***	*0.57*	*0.195*	*0.544*
**Nervonic**	**r**	−0.04	0.46	−0.02	−0.27	0.01	0.36	0.15	−0.05	0.10	−0.37
	***p***	*0.740*	*0.240*	*0.446*	*0.152*	*0.926*	*0.270*	*0.765*	*0.323*	*0.409*	*0.468*
**ω9 FAs**		**Mother Age**	**Mother Age**	**Mother Age**	**Mother Age**	**Mother Age**	**Mother Age**	**Mother Age**	**Mother Age**	**Mother Age**	**Mother Age**
**Oleic**	**r**	−0.27	−0.14	−0.24	−0.40	−0.22	−0.33	−0.01	−0.52	−0.23	−0.12
	***p***	***0.018***	*0.788*	*0.160*	***0.036***	*0.079*	*0.319*	*0.970*	***0.002***	*0.054*	*0.827*
**Gondoic**	**r**	−0.19	0.05	−0.28	−0.21	−0.22	−0.04	−0.29	−0.08	−0.14	−0.46
	***p***	*0.090*	*0.818*	*0.207*	*0.291*	*0.072*	*0.914*	*0.059*	*0.638*	*0.233*	*0.354*
**Erucic**	**r**	0.13	0.11	0.15	0.33	0.08	0.14	0.05	0.16	0.05	0.81
	***P***	*0.271*	*0.690*	*0.154*	*0.070*	*0.527*	*0.685*	*0.760*	*0.362*	*0.666*	***0.049***
**Nervonic**	**R**	0.06	0.22	−0.17	0.29	−0.03	0.23	0.03	−0.04	−0.05	0.41
	***P***	*0.583*	*0.384*	*0.612*	***0.022***	*0.813*	*0.5043*	*0.871*	*0.830*	*0.699*	*0.425*
**FAs**		**Whole Group**	**BMI Normal**	**BMI Overweight**	**BMI Obese**	**TSH Mother Normal**	**TSH Mother High**	**FT4 Mother Normal**	**FT4 Mother Low**	**TSH Newb. Normal**	**TSH Newb. High**
**Saturated FAs**		**Birth Weight**	**Birth Weight**	**Birth Weight**	**Birth Weight**	**Birth Weight**	**Birth Weight**	**Birth Weight**	**Birth Weight**	**Birth Weight**	**Birth Weight**
**Stearic**	**R**	0.02	0.11	−0.22	0.11	0.106	−0.636	−0.060	0.142	−0.02	0.77
	***P***	*0.869*	*0.188*	*0.502*	*0.696*	*0.395*	***0.035***	*0.699*	*0.422*	*0.869*	*0.072*
**Arachidic**	**R**	0.10	−0.09	0.34	−0.22	0.118	0.001	0.043	0.152	0.15	−0.71
	***P***	*0.381*	*0.823*	*0.142*	*0.216*	*0.341*	*0.998*	*0.781*	*0.392*	*0.222*	*0.111*
**Behenic**	**R**	0.24	0.69	0.20	0.09	0.293	−0.300	0.036	0.516	0.24	−0.09
	***P***	***0.036***	***0.010***	*0.886*	*0.777*	***0.016***	*0.370*	*0.816*	***0.002***	***0.038***	*0.872*
**Lignoceric**	**R**	0.27	0.64	0.17	0.17	0.329	−0.327	0.079	0.524	0.27	0.31
	***P***	***0.018***	***0.003***	*0.181*	*0.313*	***0.007***	*0.326*	*0.612*	***0.001***	***0.020***	*0.544*
**Saturated FAs**		**TSH Newb.**	**TSH Newb.**	**TSH Newb.**	**TSH Newb.**	**TSH Newb.**	**TSH Newb.**	**TSH Newb.**	**TSH Newb.**	**TSH Newb.**	**TSH Newb.**
**Stearic**	**r**	−0.24	−0.41	−0.25	0.02	−0.21	−0.523	−0.213	−0.216	−0.26	0.43
	***p***	***0.036***	*0.302*	*0.221*	*0.973*	*0.089*	*0.098*	*0.165*	*0.219*	***0.027***	*0.397*
**Arachidic**	**r**	0.05	0.64	0.02	−0.34	0.097	−0.123	0.065	0.015	0.06	0.09
	***p***	*0.680*	***0.018***	*0.658*	*0.126*	*0.435*	*0.719*	*0.673*	*0.932*	*0.595*	*0.872*
**Behenic**	**r**	0.01	−0.03	0.02	−0.12	0.019	−0.159	0.104	−0.073	−0.05	0.09
	***p***	*0.968*	*0.801*	*0.886*	*0.277*	*0.877*	*0.640*	*0.499*	*0.681*	*0.654*	*0.872*
**Lignoceric**	**r**	−0.05	−0.18	0.04	0.21	−0.03	−0.232	−0.019	−0.053	−0.08	0.77
	***p***	*0.638*	*0.407*	*0.765*	*0.975*	*0.809*	*0.492*	*0.903*	*0.766*	*0.499*	*0.072*
**Saturated FAs**		**Mother Age**	**Mother Age**	**Mother Age**	**Mother Age**	**Mother Age**	**Mother Age**	**Mother Age**	**Mother Age**	**Mother Age**	**Mother Age**
**Stearic**	**R**	0.07	0.31	0.24	−0.15	0.08	0.157	0.022	0.170	0.10	−0.12
	***P***	*0.562*	*0.307*	*0.143*	*0.622*	*0.520*	*0.645*	*0.888*	*0.336*	*0.422*	*0.827*
**Arachidic**	**R**	−0.06	0.19	−0.11	0.00	−0.139	0.346	−0.131	−0.054	−0.10	−0.14
	***P***	*0.573*	*0.535*	*0.819*	*0.987*	*0.263*	*0.298*	*0.397*	*0.762*	*0.386*	*0.784*
**Behenic**	**R**	0.00	0.04	0.12	−0.02	−0.113	−0.512	−0.244	−0.100	−0.16	0.14
	***P***	*0.989*	*0.807*	*0.284*	*0.552*	*0.361*	*0.108*	*0.110*	*0.572*	*0.177*	*0.784*
**Lignoceric**	**R**	0.03	0.02	0.17	−0.26	0.020	−0.447	−0.135	0.072	−0.02	0.06
	***P***	*0.815*	*0.943*	*0.208*	*0.662*	*0.871*	*0.168*	*0.382*	*0.685*	*0.865*	*0.913*

**r**: correlation coefficiency values, ***p***: statistical significance (written in italics). *p* values with statistical significance (<0.05) were written in bold and highlighted with pale blue.

## Data Availability

Dataset is available on request from the corresponding authors.

## Supplementary Materials

The following supporting information can be downloaded at: https://www.mdpi.com/article/10.3390/nu17122017/s1, Table S1: Summarizing table of FA correlations with birth weight, newborns’ TSH and mothers’ age.
