# Supplementary material for: ω9 Monounsaturated and Saturated Colostrum Fatty Acids May Benefit Newborns in General and Subtle Hypothyroid Stages
Source: Nutrients. 2025 Jun 17;17(12):2017. doi: 10.3390/nu17122017 (PMC12196506; doi:10.3390/nu17122017)
Supplement: Supplementary file 1 [file nutrients-17-02017-s001.zip › nutrients-3617560-supplementary.pdf]

Supplementary Table 1*. Summarizing table of FA correlations with birth weight, newborns' TSH and mothers' age

**Oleic**	**Birth Weight:** Mothers with low FT4 (positive)
	**TSH - Newborn:** Entire cohort, mothers with obesity, normal TSH, or low FT4 and newborns with normal TSH (all positive)
	**Age:** Entire cohort, mothers with obesity or low FT4 (all negative)
**Gondoic**	**Birth Weight:** Entire cohort, obese mothers, mothers with normal TSH or low FT4 and newborns with normal or high TSH (all positive)
**Erucic**	**Birth Weight:** Entire cohort, mothers with normal BMI or high TSH and newborns with normal TSH (all positive)
	**TSH - Newborn:** Mothers with obesity (negative) or normal FT4 (positive)
	**Age:** Newborns with high TSH (positive)
**Nervonic**	**Birth Weight:** Entire cohort, mothers with low FT4 and newborns with normal TSH (all positive)
	**Age:** Obese mothers (positive)
**Stearic**	**Birth Weight:** Mothers with high TSH (negative)
	**TSH - Newborn:** Entire cohort and newborns with normal TSH (negative)
**Arachidic**	**Newborns' TSH:** Mothers with normal BMI (positive)
**Behenic**	**Birth Weight:** Entire cohort, mothers with normal BMI, normal TSH, low FT4 and newborns with normal TSH (all positive)
**Lignoceric**	**Birth Weight:** Entire cohort, mothers with normal BMI, normal TSH, low FT4 and newborns with normal TSH (all positive)

*Correlations only with statistical significance are presented. Information given in brackets defines whether the observed correlations were positive or negative. Age represents the mother's age at the time of colostrum sampling.
